# Genetic characterization of the Albanian Gaucher disease patient population

**DOI:** 10.1002/jmd2.12167

**Published:** 2020-11-17

**Authors:** Paskal Cullufi, Mirela Tabaku, Virtut Velmishi, Agim Gjikopulli, Sonila Tomori, Ermira Dervishi, Aferdita Tako, Anika Leubauer, Ana Westenberger, Claudia Cozma, Christian Beetz, Peter Bauer, Stefan Wirth, Arndt Rolfs

**Affiliations:** ^1^ Pediatric Department University Hospital “Mother Teresa” Tirana Albania; ^2^ CENTOGENE GmbH Rostock Germany; ^3^ Institute of Neurogenetics University of Lübeck Lübeck Germany; ^4^ Department of Pediatrics HELIOS University Hospital Wuppertal, Centre for Clinical and Translational Research Wuppertal Germany; ^5^ Medical Faculty University of Rostock Rostock Germany

**Keywords:** Albanian population, Balkan region, Gaucher disease, *GBA*, genotype‐phenotype correlation, Lyso‐Gb1

## Abstract

Gaucher disease (GD) is a recessive metabolic disorder caused by a deficiency of the *GBA* gene‐encoded enzyme β‐glucocerebrosidase. We characterized a cohort of 36 Albanian GD patients, 31 with GD type 1 and 5 affected by GD types 2, 3, and an intermediate GD phenotype between type 2 and type 3. Of the 12 different *GBA* alleles that we detected, the most frequently observed was p.Asn409Ser, followed by p.[Asp448His;His294Gln]. The prevalence of the p.Leu483Pro allele was approximately 10‐fold lower than reported in other populations. We identified a novel pathogenic missense variant (c.1129G>A; p.Ala377Thr). All five of our non‐type 1 patients had genotypes consisting of the p.[Asp448His;His294Gln] allele in combination with another severe *GBA* allele. The median Lyso‐Gb1 level of treated patients carrying the p.[Asp448His;His294Gln] and no p.Asn409Ser allele was significantly higher than that of treated individuals homozygous or compound heterozygous for the p.Asn409Ser allele. In conclusion, the most important distinguishing features of the Albanian GD patient population are the underrepresentation of the p.Leu483Pro allele and an unusually high number of p.[Asp448His;His294Gln] alleles originating from a common Balkan founder event. The presence of at least one p.Asn409Ser allele is associated with mild disease and low Lyso‐Gb1 biomarker levels, while compound heterozygosity involving p.[Asp448His;His294Gln] and no p.Asn409Ser entails severe phenotypes and high Lyso‐Gb1 levels.

**Synposis**

In Albanian Gaucher disease (GD) patients, the frequencies of the p.[Asp448His;His294Gln] and p.Leu483Pro *GBA* alleles differ considerably from those in other populations. With respect to genotype‐phenotype correlations, the p.[Asp448His;His294Gln] allele in the absence of the “protective” p.Asn409Ser allele elicited more severe GD types and significantly higher Lyso‐Gb1 levels in comparison to p.Asn409Ser.

## 1 BACKGROUND

Gaucher disease (GD) is an autosomal recessive metabolic disorder caused by a deficiency of the lysosomal enzyme, β‐glucocerebrosidase (also called acid β‐glucosidase), responsible for the hydrolysis of the sphingolipid glucosylceramide. A decrease in β‐glucocerebrosidase activity is particularly perilous for macrophages as it leads to a progressive accumulation of glucosylceramide in their lysosomes. The swollen macrophages, termed Gaucher cells, subsequently infiltrate the liver, spleen, and bone marrow and elicit a variety of GD phenotypes.^1^, ^2^ Another consequence is the accumulation of the sphingolipid glucosylsphingosine (also known as Lyso‐Gb1), which represents both a diagnostic and monitoring biomarker.^3^, ^4^

Three main clinical types (1‐3) of GD are distinguished based on age at onset and involvement of the nervous system. While absence of neurological symptoms defines GD type 1, nervous system involvement defines types 2 and 3. Type 2 is distinguished from type 3 by onset in infancy and, if untreated, by a severe course and often fatal outcome.^5^

β‐Glucocerebrosidase is encoded by the *GBA* gene and to date, ~500 pathogenic or likely pathogenic *GBA* alleles have been reported.^6^ Of note, a difference in the distribution of pathogenic *GBA* alleles has been observed in different ethnic populations^7^, ^8^, ^9^, ^10^ and attributed to founder effect.^10^, ^11^

We investigated a large, ethnically homogeneous group of consecutively ascertained Albanian GD patients and report the types and frequencies of *GBA* alleles specific for this patient population. We also suggest a genotype‐phenotype correlation for this cohort.

## 2 PATIENTS AND METHODS

All 36 participants (including the 21 previously reported patients^12^, ^13^, ^14^) were recruited at the University Hospital Center “Mother Teresa” in Tirana, Albania. Genomic DNA was prepared from dried blood spot samples (CentoCard®). Genotyping was a three‐step procedure: (a) an initial long‐range PCR was used to specifically amplify the whole *GBA* gene (but not the *GBAP* pseudogene), (b) in a second PCR, the product from step 1 was used as a template to amplify all exons of *GBA* individually, and (c) the products from step 2 were paired‐end sequenced on an Illumina MySeq with >20× coverage for all exonic nucleotides plus the 10 neighboring intronic/UTR nucleotides. The two novel *GBA* variants were annotated and scored using PolyPhen2,^15^ SIFT,^16^ and combined annotation dependent depletion.^17^ Variant frequencies in the general population were estimated using the Genome Aggregation Database.^18^ The identified variants are labeled according to the Human Genome Variation Society recommendations.^19^ Upon the presence of two heterozygous variants, phasing was determined by checking individual reads (when both variants were in the same exon) and/or by considering the genotypes of parents (in the familial samples); in the remaining such cases, the indicated *in trans* constellation was concluded from the presence of a GD‐compatible phenotype and an increased value for the biomarker Lyso‐Gb1. Lyso‐Gb1 levels were quantified as described previously.^3^ Lyso‐Gb1 values above the cutoff of 6.8 ng/mL were considered pathological. At the time of measuring the Lyso‐Gb1 values, 24 patients were treated with taliglucerase alfa. A nonparametric Mann‐Whitney *U* test was used for statistical analysis.

## 3 RESULTS

Through the only national GD center in Albania, we identified 36 patients that had a median age at examination of 19 years (interquartile range [IQR]: 12.0‐35.5 years; range: 3 months‐68 years) (Table S1). Among these 36 individuals, 28 were unrelated index patients (4 familial and 24 sporadic; Table S2) and 8 were affected family members. Notably, two unrelated index patients formed one of the families through marriage.^14^ The three remaining families consisted of sibling pairs.

The molecular genetic testing identified 12 different *GBA* alleles in 27 of our index patients, while one variant in one index patient could not be phased. The most common *GBA* allele (A7; 42.6%) contained a p.Asn409Ser change and was found in 21 index patients (twice homozygously) and in 8 different genotypes (eg, in patients 1, 2, 5, 6, 17, and 32‐34, Table S2). The second most frequent allele (A9; 38.9%) encompassing p.Asp448His and p.His294Gln variants was identified in 20 index patients and in six genotypes (eg, in patients 6, 19, 25‐27, and 36, Table S2). All other *GBA* alleles were found in a heterozygous state in single index patients. In addition to the p.[Asp448His;His294Gln] allele, two more alleles consisted of two alterations: A8 (likely “RecΔ55 conversions”^20^), with p.Leu422Profs*4 and p.Asp448His, and A11, a recombinant allele (historically known as Rec*Nci*I) with p.Leu483Pro and p.Ala495Pro variants derived from the *GBA* pseudogene. As mentioned above, the genotype of one index patient (patient 14, Table S2) contained three changes, however, the p.Arg368His alteration could not be phased, and thus it remains unclear whether it comprises an allele with p.Asn409Ser or p.Leu483Pro. Of note, the marriage of two of our index patients (patients 31 and 32, Table S2) resulted in three affected offspring (patients 33‐35) with novel genotypes emerging from the combination of their parental alleles, as previously reported.^14^ Thus, overall, our patients harbored 15 different genotypes (Table S2).

Two of the missense variants we identified (p.Ala377Thr and p.Arg368His) were not previously reported in GD patients. The p.Ala377Thr change has been found *in trans* with another pathogenic allele and was thus classified as pathogenic (Appendix S1). The aforementioned p.Arg368His change was found *in cis* with another pathogenic variant and therefore its pathogenicity could not be assessed (Appendix S1).

**FIGURE 1:**
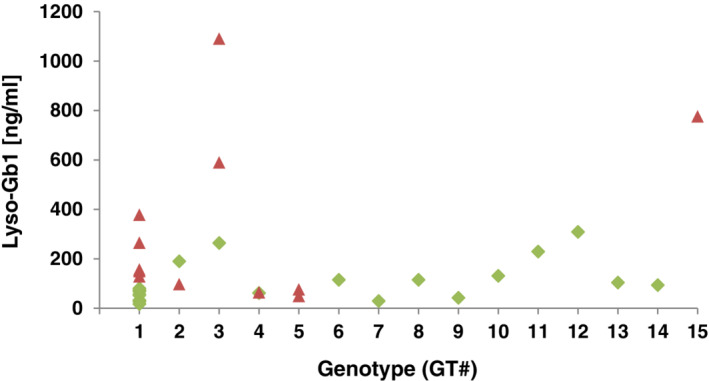
Lyso‐Gb1 values according to genotype. Measurements of Lyso‐Gb1 biomarker from dried blood spot samples for each patient are plotted against their genotype. Green diamond signs indicate that the patient was undergoing treatment at the time the sample was taken. Red triangles depict Lyso‐Gb1 values in untreated patients

The majority of our 36 patients (n = 31; 86.1%, belonging to 23 different families) were affected by GD type 1, including all of the individuals (n = 27) that carried at least one p.Asn409Ser allele (Tables S1 and S2). In all five (13.9%) of the more severely affected patients, the p.[Asp448His;His294Gln] allele was compound heterozygous with another severe *GBA* allele (GD type 3: p.Phe252Ile or p.Leu483Pro, GD type 2: c.115+1G>A, and an intermediate GD phenotype between type 2 and type 3: p.[Asp448His;His294Gln]) (Tables S1 and S2). The median Lyso‐Gb1 level in 18 treated patients with available values of this biomarker (Table S2, Figure 1) and at least one p.Asn409Ser allele (64.8 ng/mL; IQR: 32.1‐88.8 ng/mL) was significantly lower (*P* = .0188516) than the median Lyso‐Gb1 level measured in five treated individuals with at least one p.[Asp448His;His294Gln] allele and no p.Asn409Ser allele (104 ng/mL; IQR: 95.5‐249.5 ng/mL). Given that only a single individual with at least one p.[Asp448His;His294Gln] allele and no p.Asn409Ser allele (776.0 ng/mL) was untreated, we could not perform the statistical analysis between the untreated groups. However, the median Lyso‐Gb1 level in eight untreated patients with at least one p.Asn409Ser allele was 139.5 ng/mL (IQR: 67.2‐238.0 ng/mL).

Short case reports of the patients with GD types other than one are given in Appendix S1.

## 4 DISCUSSION

Our study represents a comprehensive description of the allelic spectrum of *GBA* in Albanian GD patients. Considering that there is only a single GD center in Albania, our cohort likely includes most, if not all patients diagnosed with GD in this land. Although this indicates a country‐specific prevalence of ~1 in 80 000, this figure may be an underestimate. Namely, many Albanian patients are likely misdiagnosed given that they might not be able to reach our center and therefore may not be correctly examined and treated. Studies of the frequency of GD in newborns indicate that it may be between ~1 in 15  000^21^ and ~1 in 60 000.^22^

The two most frequent alleles were p.Asn409Ser and p.[Asp448His;His294Gln] (Table 1).

**TABLE 1:** *GBA* alleles in Albanian GD patients

Allele number	Allele designation			Allele count in index patients^a^	Number of carriers (Het/Hom)
	cDNA level	Protein level	Historical nomenclature		
A1	c.115+1G>A	p.?	IVS2+1G>A	1	1/0
A2	c.256C>T	p.Arg86*	p.Arg47X	1	1/0
A3	c.259C>T	p.Arg87Trp	p.Arg48Trp	1	1/0
A4	c.437C>T	p.Ser146Leu	p.Ser107Leu	1	1/0
A5	c.754T>A	p.Phe252Ile	p.Phe213Ile	1	1/0
A6	c.1129G>A^b^	p.Ala377Thr	p.Ala338Thr	1	1/0
A7	c.1226A>G	p.Asn409Ser	p.Asn370Ser	23	19/2
A8	c.[1265_1319del;1342G>C]	p.[Leu422Profs*4;Asp448His]	55 bp deletion in exon 9 and p.Asp409His	1	1/0
A9	c.[1342G>C;882T>G]	p.[Asp448His;His294Gln]	p.[Asp409His;His255Gln]	21	19/1
A10	c.1448T>C	p.Leu483Pro	p.Leu444Pro	1	1/0
A11^c^	c.[1448T>C;1483G>C]	p.[Leu483Pro;Ala495Pro]	p.[Leu444Pro;Ala456Pro]	1	1/0
A12^d^	c.1505G>A	p.Arg502Gln*2	p.Arg463Gln*2	1	1/0

^a^ c.1103G>A (p.Arg368His) could not be phased and the index patient (patient 14; Table S2) with this variant was thus not considered above. This variant was found in patient 14 *in cis* with either a c.1226A>G or a c.1448T>C variant.

^b^ Novel variant.

^c^ A11 is also known as the recombinant allele RecNciI with the c.1448T>C and c.1483G>C variants derived from the *GBA* pseudogene.

^d^ A truncating variant first reported by Ohshima and colleagues.^32^

When considering only GD type 1 patients (n = 24), p.Asn409Ser was present in 22 (91.7%) and p.[Asp448His;His294Gln] in 16 (66.7%) individuals. Interestingly, in other studies the two alleles most frequently reported are p.Asn409Ser (75%‐100%) and p.Leu483Pro (22%‐35%) while alleles containing the p.Asp448His change were found in less than 1% to 2% of patients.^7^, ^10^, ^23^, ^24^ Thus, in the Albanian population, the frequency of patients with the p.Asn409Ser allele is comparable to that reported worldwide. In contrast, the p.Leu483Pro allele is considerably underrepresented and the p.[Asp448His;His294Gln] allele is overrepresented, with no allele harboring only the p.Asp448His variant identified among our patients. Importantly, such a high prevalence of the p.[Asp448His;His294Gln] allele in individuals of the same ethnicity indicates a shared allele/haplotype (and kinship) among all the carriers and thus corroborates a likely founder allele originating in the Balkans^11^, ^25^, ^26^ or plausibly even Albania. As a matter of fact, the low frequencies of alleles other than p.[Asp448His;His294Gln] in comparison to other populations are likely due to the commonness of this founder allele.

Two more alleles consisting of two variants each (recombinant Rec*Nci*I: p.[Leu483Pro;Ala495Pro] and likely recombinant: p.[Leu422Profs*4;Asp448His]^14^) were identified one time each.

The presence of mild *GBA* alleles such as p.Asn409Ser *in trans* with more severe alleles (eg, p.Asp448His) is known to protect patients from developing GD types with neurological involvement.^27^ Accordingly, our 18 patients carrying both p.Asn409Ser and p.[Asp448His;His294Gln] alleles manifested GD type 1. In contrast, all five of our non‐type 1 patients had genotypes consisting of the p.[Asp448His;His294Gln] allele in combination with another severe *GBA* allele (Appendix S1). Of note, the median Lyso‐Gb1 level of patients carrying the p.[Asp448His;His294Gln] and no p.Asn409Ser allele was significantly higher than that of individuals homozygous or compound heterozygous for the p.Asn409Ser allele. This finding not only confirms the neuro‐protective nature of the p.Asn409Ser allele and the severe effect of p.[Asp448His;His294Gln] at a biochemical level, but also highlights the usefulness of Lyso‐Gb1 as a biomarker in GD.^3^, ^28^ Although, our patients were treated for different lengths of time, the length of this treatment (taliglucerase alfa) does not significantly impact Lyso‐Gb1 levels, as we have recently shown.^29^

Patients homozygous for the p.[Asp448His;His294Gln] allele present with the most severe GD type 2^11^ or an intermediate GD phenotype between type 2 and type 3^30^ with early neurological impairment but slow progression of neurological symptoms.^31^ Accordingly, the boy from our cohort with the homozygous Balkan/Albania‐specific allele presented with an intermediate GD phenotype between type 2 and type 3, including gradually progressing neurological signs such as neck rigidity, head retroflexion, and oculomotor apraxia at the age of 6 months. Now, at 3 years old, his clinical condition is stable with no further neurological deterioration.

In conclusion, the most important distinguishing features of the Albanian GD patient population are the underrepresentation of the p.Leu483Pro allele, which, in contrast to p.Asn409Ser and the “Balkan allele,” is known to be a mutational hotspot, and an unusually high number of p.[Asp448His;His294Gln] alleles originating from a common Balkan founder event. The presence of at least one p.Asn409Ser allele is associated with mild disease and low Lyso‐Gb1 biomarker levels, while compound heterozygosity involving p.[Asp448His;His294Gln] and no p.Asn409Ser entails severe phenotypes and high Lyso‐Gb1 levels. Our study provides the first comprehensive insight into the clinical, genetic, and biochemical spectrum of GD in the Albanian patient population.

## CONFLICT OF INTEREST

Paskal Cullufi, Mirela Tabaku, Virtut Velmishi, Agim Gjikopulli, Sonila Tomori, Ermira Dervishi, Aferdita Tako, Anika Leubauer, and Stefan Wirth,^4^ declare that they have no conflict of interest. Ana Westenberger is a contract worker for CENTOGENE GmbH; Claudia Cozma, Christian Beetz, Peter Bauer, and Arndt Rolfs are employees of CENTOGENE GmbH.

## AUTHOR CONTRIBUTIONS

Paskal Cullufi, Claudia Cozma, Peter Bauer, Stefan Wirth, and Arndt Rolfs participated in the conception and design of the study, analysis and interpretation of data, and in the critical revision of the manuscript for important intellectual content. Mirela Tabaku, Virtut Velmishi, Agim Gjikopulli, Sonila Tomori, Ermira Dervishi, Aferdita Tako, and Anika Leubauer contributed to the analysis and interpretation of data and to the critical revision of the manuscript for important intellectual content. Ana Westenberger and Christian Beetz participated in the analysis and interpretation of data and drafting the manuscript. All authors approved the submission.

## ETHICS STATEMENT

The local ethics committee approved the study, and all the patients or their legal guardians gave written informed consent for the molecular analyses.

## Supporting information


**Appendix** **S1**. Supporting information.
