# Supplementary material for: Genetic characterization of the Albanian Gaucher disease patient population
Source: JIMD Rep. 2020 Nov 17;57(1):52–7. doi: 10.1002/jmd2.12167 (PMC7802630; doi:10.1002/jmd2.12167)
Supplement: Supplementary file 1 — Appendix S1. Supporting information. [file JMD2-57-52-s001.docx]

**SUPPLEMENTARY MATERIAL**

**Genetic characterization of the Albanian Gaucher disease patient population**

**Supplementary Results**

*Case reports*

Patient 36 (GD type 2; c.[115+1G>A];p.[Asp448His;His294Gln])

This male infant was diagnosed with type 2 GD at the age of 2 months. He manifested with a severe syndrome with visceral and neurological involvement and enlargement of the liver and spleen. In addition, he had cholestasis, trunk spasticity and oculomotor disorder. The patient passed away at the age of 3 months.

Patients 19 and 28 (GD type 3; p.[Phe252Ile];[Asp448His;His294Gln])

Patient 19 is a 25-year old man, affected by GD type 3 and manifesting with seizure, oculomotor disorders, and walk and speech difficulties in combination with emotional lability and other symptoms of mood disorder. His phenotype is more severe than the clinical presentation of his 15-year old sister (Patient 28).

Patient 26 (GD type 3; p.[Leu483Pro];[Asp448His;His294Gln])

This 4-year-old boy suffers from GD type 3 with oculomotor apraxia as the only symptom of neurological involvement.

Patient 25 (GD type 2-3; [Asp448His;His294Gln];[Asp448His;His294Gln])

This patient is currently a 3-year-old boy, who presented at the age of 6 months with a phenotype that was not typical of either acute type 2 GD or of subacute type 3 neuronopathic form. At that time, his clinical manifestation included severe hepatosplenomegaly and gradually progressing neurological signs such as neck rigidity, head retroflelxion, and oculomotor apraxia. Now, at the age of 3, he is being treated with velaglucerase alfa, and his clinical condition is stable with improvement of visceral signs and no further neurological deterioration.

*Scoring and classification of novel variants*

**c.1103G>A (p.Arg368His) (CADD score: 17.9)**

The GBA variant c.1103G>A (p.Arg368His) affects a moderately conserved amino acid. The amino acids arginine and histidine are both polar and their physicochemical properties do not differ considerably. Software analyses showed likely benign predictions. The gnomAD database lists this variant with a frequency in the general population of 0.0065% (16 heterozygous carriers); however, this variant was never reported in GD or Parkinson’s disease (PD) patients. A different amino acid change at the same position (p.Arg368Cys) has been reported as disease causing for PD ^1^. Importantly, this variant was found in cis with another pathogenic variant (either p.Asn409Ser or p.Leu483Pro; GT10 in Table 1B) and therefore its pathogenicity could not be determined.

**c.1129G>A (p.Ala377Thr) (CADD score: 25.8)**

The GBA variant c.1129G>A (p.Ala377Thr) affects a moderately conserved amino acid. The amino acids alanine and threonine differ in their side chain polarity (nonpolar and polar, respectively). Software analyses indicated a possibly disease-causing effect. To date, this variant has not been reported in gnomAD or in GD or PD patients. A deleterious effect of this variant has been confirmed by *in vivo* measurements of biomarker levels [i.e., Lyso-Gb1]. This collection of evidence indicates that the c.1129G>A change is pathogenic.

**Supplementary Table 1.** Demographic and clinical structure of the investigated GD patient population.

| Number of patients | | 36 |
| --- | --- | --- |
| Ethnicity (race) | | Albanian (Caucasian) |
| Gender | | 22 (61%) male;  14 (39%) female |
| Patient younger than 18 years | | 16 (44.4%) |
| Type 1 GD | Number (percentage) | 31 (86.1%) |
|  | Gender | 18 male/13 female |
|  | Median age (IQR) | 21 (13-37) years |
| Type 2 GD | Number (percentage) | 1 (2.8%) |
|  | Gender | male |
|  | Age | 3 months |
| Intermediate GD phenotype (between types 2 and 3) | Number (percentage) | 1 (2.8%) |
|  | Gender | male |
|  | Age | 13 months |
| Type 3 GD | Number (percentage) | 3 (8.3%) |
|  | Gender | 2 male/1 female |
|  | Ages | 4, 15, and 25 years |

GD - Gaucher disease; IQR - Interquartile range; Age refers to the age at the time of last examination and of Lyso-Gb1 measurement.

**Supplementary Table 2.** Clinical diagnoses, Lyso-Gb1 measurements, and genotypes of the investigated patients.

| **Family ID** | **Patient ID** | **Gender** | **GD type** | **Shehi et al. 2011**^2^ | **Velmishi et al. 2013**^3^ | **Cullufi et al. 2019**^4^ | **Lyso-Gb1 [ng/ml] and treatment status** | **Age*** | **Genotypes** |
| --- | --- | --- | --- | --- | --- | --- | --- | --- | --- |
| F1 | 6^§**^ | Female | 1 | n.r. | P18 | n.r. | 68.9 (treated) | 37 years | p.Asn409Ser/p.[Asp448His;His294Gln] |
|  | 12^§^ | Male | 1 | n.r. | P19 | n.r. | 52.8 (treated) | 34 years | p.Asn409Ser/p.[Asp448His;His294Gln] |
| F2 | 13^§^ | Female | 1 | n.r. | P16 | n.r. | 56.7 (treated) | 9 years | p.Asn409Ser/p.[Asp448His;His294Gln] |
|  | 15^§**^ | Male | 1 | n.r. | P17 | n.r. | 32.8 (treated) | 13 years | p.Asn409Ser/p.[Asp448His;His294Gln] |
| F3 | 19^§**^ | Male | 3 | P3 | P3 | n.r. | 97.5 (treated) | 25 years | p.Phe252Ile/p.[Asp448His;His294Gln] |
|  | 28 | Female | 3 | n.r. | n.r. | n.r. | 190.0 (treated) | 15 years | p.Phe252Ile/p.[Asp448His;His294Gln] |
| F4 | 21^§**^ | Male | 1 | n.r. | n.r. | I-3 | 590.0 (untreated) | 36 years | p.Arg87Trp/p.[Leu422Profs*4;Asp448His] |
|  | 23^§^ | Female | 1 | n.r. | n.r. | I-2 | 1090.0 (untreated) | 40 years | p.Arg87Trp/p.[Leu422Profs*4;Asp448His] |
|  | 31^§^ | Male | 1 | n.r. | n.r. | I-1 | 264.0 (treated) | 43 years | p.Arg87Trp/p.[Leu422Profs*4;Asp448His] |
|  | 32^§**^ | Female | 1 | n.r. | n.r. | I-4 | 64.2 (untreated) | 43 years | p.Asn409Ser/p.Asn409Ser |
|  | 33^§^ | Male | 1 | n.r. | n.r. | II-2 | 49.6 (untreated) | 14 years | p.Arg87Trp/p.Asn409Ser |
|  | 34^§^ | Male | 1 | n.r. | n.r. | II-1 | 115.0 (treated) | 17 years | p.Asn409Ser/p.[Leu422Profs*4;Asp448His] |
|  | 35^§^ | Female | 1 | n.r. | n.r. | II-3 | 76.3 (untreated) | 7 years | p.Arg87Trp/p.Asn409Ser |
| F5 | 1^§**^ | Female | 1 | P8 | P8 | n.r. | 29.5 (treated) | 21 years | p.Asn409Ser/p.Arg502Gln*2 |
| F6 | 2^§**^ | Male | 1 | P6 | P6 | n.r. | 115.0 (treated) | 13 years | p.Asn409Ser/p.Arg86* |
| F7 | 3^**^ | Male | 1 | n.r. | n.r. | n.r. | 73.5 (treated) | 56 years | p.Asn409Ser/p.[Asp448His;His294Gln] |
| F8 | 4^§**^ | Female | 1 | P4 | P4 | n.r. | 72.7 (treated) | 21 years | p.Asn409Ser/p.[Asp448His;His294Gln] |
| F9 | 5^**^ | Male | 1 | n.r. | n.r. | n.r. | 42.4 (treated) | 28 years | p.Asn409Ser/p.[Leu483Pro;Ala495Pro] |
| F10 | 7^**^ | Female | 1 | n.r. | n.r. | n.r. | 150.0 (untreated) | 19 years | p.Asn409Ser/p.[Asp448His;His294Gln] |
| F11 | 8^§**^ | Male | 1 | P5 | P5 | n.r. | 27.0 (treated) | 19 years | p.Asn409Ser/p.[Asp448His;His294Gln] |
| F12 | 9^§**^ | Male | 1 | P10 | P10 | n.r. | 29.8 (treated) | 13 years | p.Asn409Ser/p.[Asp448His;His294Gln] |
| F13 | 10^§**^ | Female | 1 | n.r. | P15 | n.r. | 67.7 (treated) | 32 years | p.Asn409Ser/p.[Asp448His;His294Gln] |
| F14 | 11^**^ | Male | 1 | n.r. | n.r. | n.r. | 61.8 (treated) | 29 years | p.Asn409Ser/p.Asn409Ser |
| F15 | 14^§**^ | Male | 1 | P9 | P9 | n.r. | 131.0 (treated) | 12 years | p.Asn409Ser/p.Leu483Pro ^#^p.Arg368His |
| F16 | 16^§**^ | Male | 1 | n.r. | P14 | n.r. | 80.1 (treated) | 13 years | p.Asn409Ser/p.[Asp448His;His294Gln] |
| F17 | 17^§**^ | Female | 1 | P7 | P7 | n.r. | 229.0 (treated) | 16 years | p.Asn409Ser/p.Ser146Leu |
| F18 | 18^**^ | Male | 1 | n.r. | n.r. | n.r. | 16.7 (treated) | 43 years | p.Asn409Ser/p.[Asp448His;His294Gln] |
| F19 | 20^**^ | Male | 1 | n.r. | n.r. | n.r. | 378.0 (untreated) | 68 years | p.Asn409Ser/p.[Asp448His;His294Gln] |
| F20 | 22^**^ | Female | 1 | n.r. | n.r. | n.r. | 129.0 (untreated) | 54 years | p.Asn409Ser/p.[Asp448His;His294Gln] |
| F21 | 24^**^ | Female | 1 | n.r. | n.r. | n.r. | 157.0 (untreated) | 7 years | p.Asn409Ser/p.[Asp448His;His294Gln] |
| F22 | 25^**^ | Male | 2-3 | n.r. | n.r. | n.r. | 309.0 (treated) | 1 years | p.[Asp448His;His294Gln]/  p.[Asp448His;His294Gln] |
| F23 | 26^**^ | Male | 3 | n.r. | n.r. | n.r. | 104.0 (treated) | 4 years | p.Leu483Pro/p.[Asp448His;His294Gln] |
| F24 | 27^**^ | Male | 1 | n.r. | n.r. | n.r. | 93.5 (treated) | 3 years | p.Ala377Thr/p.[Asp448His;His294Gln] |
| F25 | 29^**^ | Male | 1 | n.r. | n.r. | n.r. | n.a. | 21 years | p.Asn409Ser/p.[Asp448His;His294Gln] |
| F26 | 30^**^ | Female | 1 | n.r. | n.r. | n.r. | 265.0 (untreated) | 12 years | p.Asn409Ser/p.[Asp448His;His294Gln] |
| F27 | 36^**^ | Male | 2 | n.r. | n.r. | n.r. | 776.0 (untreated) | 2 months | c.115+1G>A/p.[Asp448His;His294Gln] |

^§^ - previously reported; * - at the time of Lyso-Gb1 measurement; ^#^ - c.1103G>A (p.Arg368His) could not be phased and it is thus positioned in cis with either c.1226A>G or c.1448T>C variant in Patient 14; ^**^ - index patient; n.a. – not available; n.r. – not reported.

**Supplementary References**

1. Lwin A, Orvisky E, Goker-Alpan O, et al. Glucocerebrosidase mutations in subjects with parkinsonism. [Internet]. Mol Genet Metab 2004;81(1):70–3.[cited 2019 Sep 18 ] Available from: http://www.ncbi.nlm.nih.gov/pubmed/14728994

2. Shehi B, Boçari G, Vyshka G, et al. Gaucher’s disease in Albanian children: Casuistics and Treatment [Internet]. Iran J Pediatr 2011;21(1):1–7.[cited 2020 Aug 23 ] Available from: https://pubmed.ncbi.nlm.nih.gov/23056756/

3. Velmishi V. Clinical and Diagnostic Findings of 19 Gaucher Patients in Albania. J Liver 2013;02(02):1–4.

4. Cullufi P, Tabaku M, Beetz C, et al. Comprehensive clinical, biochemical and genetic screening reveals four distinct GBA genotypes as underlying variable manifestation of Gaucher disease in a single family. [Internet]. Mol Genet Metab reports 2019;21:100532.[cited 2020 Mar 9 ] Available from: http://www.ncbi.nlm.nih.gov/pubmed/31709146
